# 

**Published:** 1889-03

**Authors:** 


					﻿io cts. a copy
MARCH, .1889.
$ i .exo per } ear.
HALLS
eJoviVN/XL'J
HEALTH
ESTABLISHED 18^4
U°k- 36
No. 3.
OFFICE: 206 BROADWAY. EVENING POST BUILDING, Boom ll.N. Y.
Entered as Second-class Matter at the Post-Office, New York.
				

